# Long-Term Citalopram Treatment Alters the Stress Responses of the Cortical Dopamine and Noradrenaline Systems: the Role of Cortical 5-HT_1A_ Receptors

**DOI:** 10.1093/ijnp/pyw026

**Published:** 2016-03-30

**Authors:** Fumi Kaneko, Yukie Kawahara, Yuki Kishikawa, Yuuki Hanada, Makiko Yamada, Tatsuyuki Kakuma, Hiroshi Kawahara, Akinori Nishi

**Affiliations:** Department of Pharmacology, Kurume University School of Medicine, Kurume, Fukuoka, Japan (Ms Kaneko and Drs Kawahara, Kishikawa, Hanada, and Nishi); Department of Psychiatry, Tokyo Women’s Medical University, Shinjuku-ku, Tokyo, Japan (Dr Yamada); Biostatistics Center, Kurume University, Kurume, Fukuoka, Japan (Dr Kakuma); Department of Dental Anesthesiology, School of Dentistry, Tsurumi University, Tsurumi-ku, Yokohama, Kanagawa, Japan (Dr Kawahara).

**Keywords:** citalopram, catecholamine, 5-HT_1A_ receptor, microdialysis, prefrontal cortex

## Abstract

**Background:**

Cortical dopamine and noradrenaline are involved in the stress response. Citalopram, a selective serotonin reuptake inhibitor, has direct and indirect effects on the serotonergic system. Furthermore, long-term treatment with citalopram affects the dopamine and noradrenaline systems, which could contribute to the therapeutic action of antidepressants.

**Methods:**

The effects of long-term treatment with citalopram on the responses of the dopamine and noradrenaline systems in the rat prefrontal cortex to acute handling stress were evaluated using in vivo microdialysis.

**Results:**

Acute handling stress increased dopamine and noradrenaline levels in the prefrontal cortex. The dopamine and noradrenaline responses were suppressed by local infusion of a 5-HT_1A_ receptor agonist, 7-(Dipropylamino)-5,6,7,8-tetrahydronaphthalen-1-ol;hydrobromide, into the prefrontal cortex. The dopamine response was abolished by long-term treatment with citalopram, and the abolished dopamine response was reversed by local infusion of a 5-HT1_A_ receptor antagonist, (Z)-but-2-enedioic acid;N-[2-[4-(2-methoxyphenyl)piperazin-1-yl]ethyl]-N-pyridin-2-ylcyclohexanecarboxamide into the prefrontal cortex. On the other hand, long-term treatment with citalopram reduced the basal noradrenaline levels (approximately 40% of the controls), but not the basal dopamine levels. The noradrenaline response was maintained despite the low basal noradrenaline levels. Signaling from the 5-HT_1A_ receptors and α_2_-adrenoceptors was not involved in the decrease in the basal noradrenaline levels but partially affected the noradrenaline response.

**Conclusions:**

Chronic citalopram treatment differentially suppresses the dopamine and noradrenaline systems in the prefrontal cortex, and the dopamine stress response was preferentially controlled by upregulating 5-HT_1A_ receptor signaling. Our findings provide insight into how antidepressants modulate the dopamine and noradrenaline systems to overcome acute stress.

## Introduction

The mesocortical dopamine (DA) neurons are highly sensitive to stressful stimuli (Thierry et al., 1976; Segovia et al., 2009). Among subpopulations of DA neurons in the ventral tegmental area (VTA), the mesocortical DA neurons are unique, as they are excited by aversive stimuli but not by rewarding stimuli (Lammel et al., 2011). The DA levels in the prefrontal cortex (PFC) increase in response to various types of acute stress, such as handling stress (Enrico et al., 1998; Takahata and Moghaddam, 1998; Y. Kawahara et al., 1999; Feenstra et al., 2000; Del Arco and Mora, 2001), restraint stress (Cuadra et al., 2001; Mokler et al., 2007; Ahmad et al., 2012), foot shock (Bannon and Roth, 1983), and acute social defeat (Tanaka et al., 2012). Handling stress has been used as a mild acute stressor, because handling is a routine laboratory procedure and is obviously less stressful compared with severe stressors with pain and fear, resulting in the maladaptive state of homeostasis called distress (Balcombe et al., 2004). Circulating corticosterone is a sensitive index for evaluating the stress intensity and correlates with stress intensities at low and middle levels (Armario et al., 1986; De Boer et al., 1990). As proof of handling stress for the mild stressor, the handling stress-induced increase in circulating corticosterone (2- to 4-fold) is equivalent to the increase by novelty stress (2- to 5-fold) but smaller than the increases by tail pinch (5-fold), cold exposure (6-fold), restraint (7- to 32-fold), and water immersion (9- to 14-fold) stresses (Armario et al., 1986; De Boer et al., 1990; Balcombe et al., 2004; de Oliveira et al., 2004; Butts et al., 2011).

Acute stressful stimuli induce an increase in the noradrenaline (NA) levels in various brain regions (Y. Kawahara et al., 1999; Morilak et al., 2005). In addition, rewarding and stressful stimuli increase the NA levels in the PFC (Feenstra et al., 2000; Ihalainen and Tanila, 2002; Mingote et al., 2004; Y. Kawahara et al., 2007; Ventura et al., 2007). The concomitant increases in the DA and NA levels in the PFC in response to acute stressful stimuli might be important to process the stress.

The serotonergic system is implicated in the pathophysiology of depression and anxiety, and a selective serotonin reuptake inhibitor (SSRI), which increases serotonergic neurotransmission via inhibition of serotonin reuptake, is commonly used to treat depression and anxiety (Hoffman and Mathew, 2008; Holmes, 2008; Serretti et al., 2011; Albert et al., 2014). Although serotonergic modulation of the responses to acute and chronic stress is mediated through many molecules, the 5-HT_1A_ receptor is one of key components (Richardson-Jones et al., 2010; Albert et al., 2014). In the PFC, 5-HT_1A_ receptors are abundantly expressed as postsynaptic heteroreceptors on 2 neuronal populations, excitatory pyramidal neurons and inhibitory GABAergic interneurons (Amargos-Bosch et al., 2004; Santana et al., 2004), and are known to regulate DA neurotransmission (Rasmusson et al., 1994; Wedzony et al., 1996; Llado-Pelfort et al., 2012). The 5-HT_1A_ heteroreceptor as well as the 5-HT_1A_ autoreceptor expressed on raphe serotonergic neurons plays significant roles in the stress responses and anxiety- and depression-like behaviors, as demonstrated in various genetic 5-HT_1A_ receptor models (e.g., knockout, suppression, or overexpression of auto- and hetero-receptors) (Albert et al., 2014). In humans, 5-HT_1A_ partial agonists, such as buspirone, are used to treat anxiety-related disorders (Ettenberg and Bernardi, 2006; Graeff and Zangrossi, 2010). Furthermore, signaling through the postsynaptic 5-HT_1A_ receptor has recently been suggested as a target for antidepressants (Celada et al., 2013).

Citalopram, one of the first-line SSRIs, is used to treat depression (Cipriani et al., 2012) and anxiety disorders (Davidson, 2009). We previously reported that chronic citalopram treatment attenuates the NA response to handling stress in the basolateral amygdala due to sensitization of α_2_-adrenoceptors (Y. Kawahara et al., 2007). Thus, the monoaminergic network, including the serotonin, DA, and NA pathways, is highly interconnected, and the interconnection might be modulated by chronic antidepressant treatment (Hamon and Blier, 2013). In this study, we investigated the effect of chronic citalopram treatment on the DA and NA systems in the PFC and found that chronic citalopram treatment differentially affected the responses of the DA and NA systems to acute handling stress. The mechanisms for the altered responses were further investigated.

## Methods

### Animals

Male albino Wistar rats (280–320g, Kyudo, Tosu, Japan) were maintained at 23±2°C under a 12-hour-light/-dark cycle with free access to food and water. Two rats were housed in one home cage until surgery. All rats used in this study were handled in accordance with the Guide for the Care and Use of Laboratory Animals as adopted and promulgated by the US National Institutes of Health, and the specific protocols were approved by the Committee for Animal Experimentation, Kurume University School of Medicine. All efforts were made to minimize the number of animals used.

### Drugs

1-[3-(Dimethylamino)propyl]-1-(4-fluorophenyl)-1,3-dihydro-2-benzofuran-5-carbonitrile; hydrobromide (citalopram hydrobromide) was generously supplied by H. Lundbeck (A/S Copenhagen, Denmark) and was dissolved in saline (0.2mL) and Ringer’s solution for systemic injection and local infusion, respectively. 7-(Dipropylamino)-5,6,7,8-tetrahydronaphthalen-1-ol;hydrobromide (8-OH-DPAT hydrobromide), (±)-2-(2,3-dihydro-1,4-benzodioxin-2-yl)-4,5-dihydro-1H-imidazole;hydrochloride (idazoxan hydrochloride), and (Z)-but-2-enedioic acid;N-[2-[4-(2-methoxyphenyl)piperazin-1-yl]ethyl]-N-pyridin-2-ylcyclohexanecarboxamide (WAY-100,635 maleate salt) were purchased from Sigma-Aldrich Co. (St. Louis, MO) and were dissolved in Ringer’s solution for local infusion.

### Surgery and Brain Dialysis

Microdialysis was performed with an I-shaped cannula. The probes were implanted in the right PFC (exposed length 5mm) under pentobarbital (50mg/kg i.p.) and xylazine (8mg/kg i.p.) anesthesia and a local application of 10% lidocaine. The coordinates of the implantation were A/P +2.5mm, L/M 2.0mm from the bregma, and V/D 6.0mm from the dura at an angle of 14° in the coronal plane. After probe implantation, the rats were housed individually in plastic cages (30×30×40cm). The microdialysis experiments were conducted 24 hours after implantation of the probes, as previously described (Kawahara et al. 2007, 2009). An online approach was used in which the probes were perfused with Ringer’s solution at a flow rate of 2 µL/min through an infusion pump (EICOM, Kyoto, Japan). The dialysate fractions were collected every 20 minutes. The DA and NA levels were quantified by high-performance liquid chromatography using a reverse-phase column (150×4.6mm; Supelco LC18, Bellefonte, PA) with electrochemical detection. An EP-300 pump (EICOM) was used in conjunction with an electrochemical detector (ESA; potential of the first cell, +180 mV; potential of the second cell, -180 mV). The mobile phase was a mixture of 4.1g/L sodium acetate adjusted to pH 5.5, 50mg/L Na_2_EDTA, 140mg/L octanesulfonic acid, and 10% v/v methanol. The flow rate was 0.6mL/min. The detection limit of the assay was approximately 0.3fmol per sample (on-column). The composition of the Ringer’s solution was (in mM): NaCl 140.0, KCl 4.0, CaCl_2_ 1.2, and MgCl_2_ 1.0. After collection of 3 baseline samples, the animals were subjected to handling stress. After the experiments, the rats were given an overdose of chloral hydrate, and their brains were fixed with 4% paraformaldehyde via intracardiac infusion. Coronal sections (16 μm thick) were cut, and dialysis probe placement was localized using the atlas of Paxinos and Watson (2013). The rats in which the dialysis probes and guide cannula were misplaced were not included in the data analysis.

### Administration of Citalopram

The rats in the chronic vehicle-treated and citalopram-treated groups were treated with saline (0.2mL s.c.) and citalopram (10mg/kg in 0.2mL saline s.c.) once daily for 14 days, respectively. Brain microdialysis was conducted 48 hours after the last injection. For the acute treatment, citalopram (10mg/kg in 0.2mL saline s.c.) was administered 40 minutes before application of handling stress.

### Forced Swim Test

The method of Slattery and Cryan (2012) was used to assess the immobility of rats as a measure of their helplessness or depressive-like behavior. After 26 to 27 hours of isolation following surgery for microdialysis probe implantation, rats were placed individually in a round Pyrex cylinder pool measuring 28.0cm in diameter and 45.5cm in height for 5 minutes. The cylinder was filled with 30cm of water (25±1°C) to ensure that animals could not touch the bottom of the container with their hind paws or tails. Fresh water was used for each forced swim test (FST) in every animal. Immobility was defined as no additional activity other than that required to keep the head above water.

### Stress Exposure

A rat was removed from the home cage and held in the hand for 20 minutes using latex gloves covered by cotton work gloves to produce mild emotional stress. The stress was applied during light phase between 2:00 and 4:00 pm. The rats were mildly immobilized for the first couple of minutes since the rats struggled to escape from the hands. When rats started staying quietly in hands, they were kept as they were without any additional treatment (e.g., stroking) unless they struggled to escape.

### Data and Statistics

**Figure 1.**
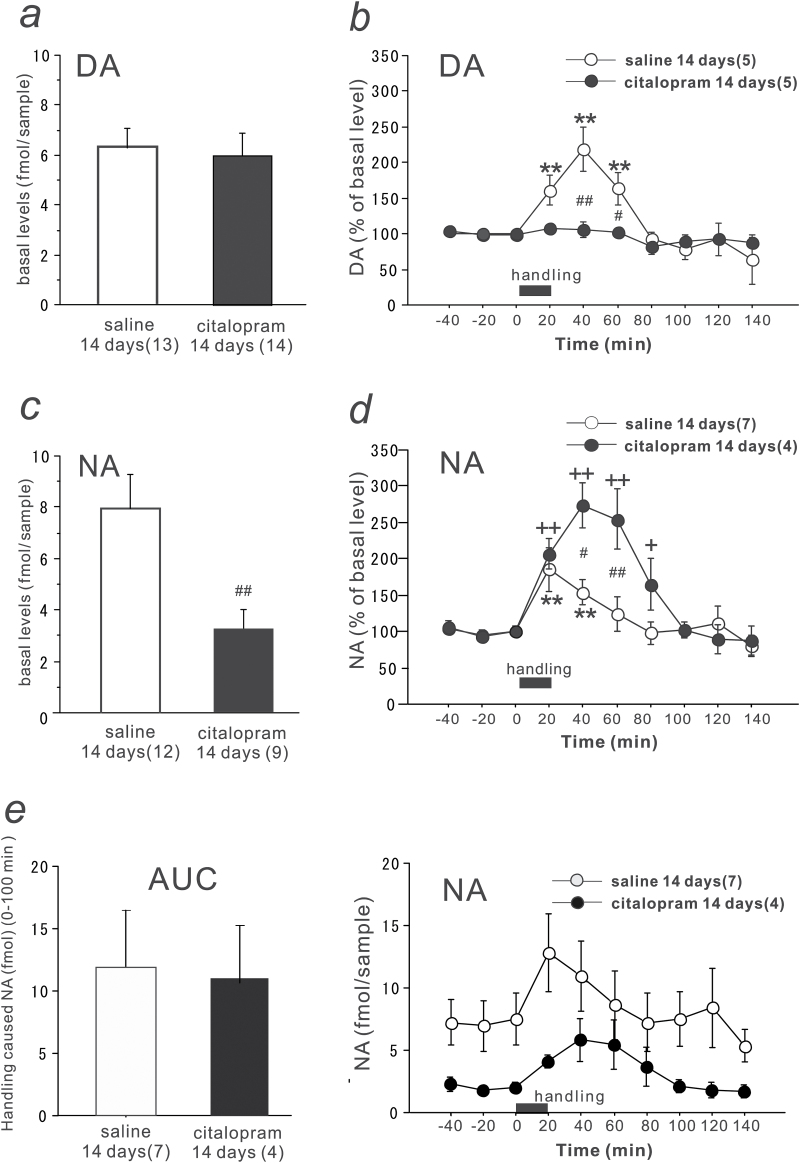
Effects of long-term citalopram administration on the basal and handling stress-induced dopamine (DA) and noradrenaline (NA) levels in dialysates from the prefrontal cortex (PFC). (a, c) The basal extracellular levels of DA (a) and NA (c) in the PFC were determined with in vivo microdialysis in rats treated with saline or citalopram (10mg kg/d, s.c.) for 14 days. (b, d) Effects of handling stress on the extracellular levels of DA (b) and NA (d) in the PFC in rats treated with saline (open circles) or citalopram (closed circles) for 14 days. All values are calculated as a percentage of the basal values within the same group (100%). (e) The absolute values of the handling stress-induced increases in the NA in rats treated with saline (open circles) or citalopram (closed circles). **All rats received an infusion of Ringer’s solution into the PFC as the perfusate of the microdialysis probes.** The closed squares indicate the 20-minute handling period. (left) The comparison of area under the curve (AUC) for the increase in NA above the basal levels after handling stress (0–100 minutes). The numbers of experiments are shown in parentheses. The data are expressed as the means ± SEM. **P < .01 vs the basal levels of the saline-treated group; ^+^ *P* < .05, ^++^ *P* < .01 vs the basal levels of the citalopram-treated group; ^#^ *P* <.05, ^##^ *P* < .01 vs the saline-treated group.

An unpaired *t* test was used to compare the basal DA and NA levels in the saline- and citalopram-treated groups, and 1-way ANOVA and Dunnet’s test for post hoc determination were used to compare the immobility time in the FST (JMP Pro, SAS Institute, Cary, NC). The DA and NA levels in the drug-infused group were obtained as the average of 3 samples during the 1 to 2 hours of the drug infusion period. All values, except absolute values in Figure 1e, were expressed as a percentage of the basal values (100%), obtained as the average of 3 stable baseline samples. The values obtained after handling stress were compared with the basal values using mixed linear models with the measurement time as a covariate, and the details of the statistical analysis are listed in supplementary Table 1. Bonferroni’s correction was applied for multiple comparisons using the SAS MIMED procedure (version 9.4, SAS Institute). Repeated-measures 2-way ANOVA and Tukey’s HSD test for posthoc determination were used to compare the experimental groups (JMP Pro). The area under the curve (AUC) was presented as the total absolute amount of NA increased above the basal levels after handling stress for 0 to 100 minutes. The level of significance was set at *P* < .05.

## Results

### Effects of Long-Term Citalopram Administration on the Basal and Handling Stress-Induced DA and NA Levels in Dialysates from the PFC

Daily citalopram (10mg kg/d, s.c.) treatment for 14 days did not affect **the basal levels of DA** in the rat PFC (Figure 1a) **but decreased the basal levels of NA in the PFC to approximately 40% of the level in the saline-treated rats** [t(19) = -3.090, *P* = .0060] (Figure 1c).

Twenty minutes of handling stress induced a maximal increase in the DA levels to 220% of the basal levels at 40 minutes in the saline-treated rats (Figure 1b). The handling stress-induced increase in the DA levels was not observed in the citalopram-treated rats (group effect, F_(1, 76)_ = 11.2249, *P* < .0013; time effect, F_(9, 76)_ = 7.4931, *P* < .0001; group-time interaction, F_(9, 76)_ = 5.3786, *P* < .0001). On the other hand, handling stress induced increases in the NA levels in both the saline- and citalopram-treated rats (Figure 1d). The relative increase in the NA levels in the citalopram-treated rats was larger than that in the saline-treated rats (270% vs 190% of the basal levels) (group effect, F_(1, 90)_ = 12.4769, *P* = .0007; time effect, F_(9, 90)=_11.3617, *P* < .0001; group-time interaction, F_(9, 90)_ = 3.487, *P* = .001). When absolute values of NA contents were analyzed (group effect, F_(1, 90)_ = 23.5569, *P* < .0001; time effect, F_(9, 90)_ = 1.0154, *P* = .4339; group-time interaction, F_(9, 90)_ = 0.2451, *P* = .9866) (Figure 1e), the increases in NA above the basal levels after handling stress, expressed as AUC (0–100 minutes), were similar in the saline- and citalopram-treated rats. The results suggest that the handling stress-induced increases in NA were not altered after long-term citalopram treatment, despite the substantial decrease in the basal NA levels.

Acute administration of citalopram (10mg kg/d, s.c.) did not affect the basal levels of DA and NA in the PFC (supplementary Figure 1a). When handling stress was applied 40 minutes after a single administration of citalopram, handling stress induced increases in DA levels (group effect, F_(1, 66)_ = 9.6780, *P* = .0028; time effect, F_(10, 66)_ = 6.8943, *P* < .0001; group-time interaction, F_(10, 66)_ = 3.9415, *P* = .0003) and NA levels (group effect, F_(1, 66)_ = 10.9382, *P* = .0015; time effect, F_(10, 66)_ = 6.3850, *P* < .0001; group-time interaction, F_(10, 66)_ = 2.9755, *P* = .0038). The responses of DA and NA were similar to those in the chronically saline-treated rats. The results suggest that long-term treatment of citalopram is required to elicit its effects on the DA and NA systems.

### Effects of a Local Infusion of a 5-HT_1A_ Receptor Agonist, 8-OH-DPAT, into PFC of Naïve Rats on Handling Stress-Induced Increases in DA and NA Levels

**Figure 2.**
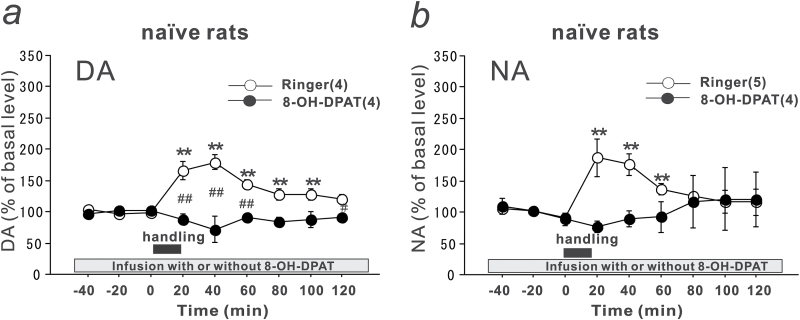
Effects of a local infusion of a 5-HT_1A_ receptor agonist, 7-(Dipropylamino)-5,6,7,8-tetrahydronaphthalen-1-ol;hydrobromide (8-OH-DPAT), into the prefrontal cortex (PFC) of naïve rats on the handling stress-induced increases in the dopamine (DA) and noradrenaline (NA) levels in dialysates from the PFC. Naïve rats received a local infusion of Ringer’s solution (open circles) or 8-OH-DPAT (10 µM) (closed circles) into the PFC, and the effects of handling stress on the extracellular levels of (a) DA and (b) NA in the PFC were examined. The closed squares indicate the 20-minute handling period. All values are calculated as a percentage of the basal values within the same group. The numbers of experiments are shown in parentheses. The data are expressed as the means ± SEM. **P < .01 vs the basal levels of the Ringer’s solution-infused group; ^##^ *P* < .01 vs the Ringer’s solution-infused group.

To investigate the mechanisms by which chronic citalopram treatment suppresses the handling stress-induced increase in the DA levels, the effects of handling stress were examined in naïve rats, which received an infusion of Ringer’s solution or a 5-HT_1A_ receptor agonist, 8-OH-DPAT (10 µM), into the PFC. Local infusion of this dose of 8-OH-DPAT for 1 to 2 hours did not affect either the basal DA or NA levels (97.32±8.42% and 91.69±8.77% of the basal levels, respectively). Twenty minutes of handling stress induced an increase in the DA levels to 180% of the basal levels in the Ringer’s solution-infused rats (Figure 2a). In contrast, the handling stress-induced increase in the DA levels was not observed in the 8-OH-DPAT–infused rats. The results demonstrate that activation of the 5-HT_1A_ receptors suppresses the DA responses to handling stress (group effect, F_(1, 61)_ = 81.941, *P* < .0001; time effect, F_(8, 61)_ = 2.9455, *P* = .0075; group-time interaction, F_(8, 61)_ = 10.1536, *P* < .0001), as observed after chronic citalopram treatment.

Handling stress induced an increase in the NA levels to 190% of the basal levels in the Ringer’s solution-infused rats (Figure 2b). The infusion of 8-OH-DPAT into the PFC also suppressed the handling stress-induced increase in the NA levels (group effect, F_(1,54)_ = 6.103, *P* = .0167; time effect, F_(8, 54)_ = 0.7968, *P* = .6078; group-time interaction, F_(8, 54)_ = 1.8946, *P* = .0798).

### Effects of a Local Infusion of a 5-HT_1A_ Receptor Antagonist, WAY-100,635, into the PFC of Citalopram-Treated Rats on the DA and NA Levels after Handling Stress

**Figure 3.**
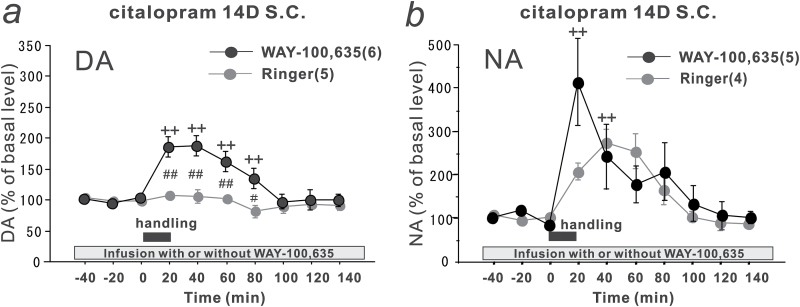
Effects of a local infusion of a 5-HT_1A_ receptor antagonist, Z)-but-2-enedioic acid;N-[2-[4-(2-methoxyphenyl)piperazin-1-yl]ethyl]-N-pyridin-2-ylcyclohexanecarboxamide (WAY-100,635) into the prefrontal cortex (PFC) of citalopram-treated rats on the dopamine (DA) and noradrenaline (NA) levels after handling stress. The citalopram-treated rats (10mg kg/d s.c. for 14 days) received a local infusion of WAY-100,635 (1 µM) (closed circles) into the PFC, and the effects of handling stress on the extracellular levels of DA (a) and NA (b) in the PFC were compared with those in citalopram-treated rats infused with Ringer’s solution (gray circles, reproduced from Figure 1b and 1d for comparison). The closed squares indicate the 20-minute handling period. All values are calculated as a percentage of the basal values within the same group. The numbers of experiments are shown in parentheses. The data are expressed as the means ± SEM. ^++^ *P* < .01 vs. the basal levels of the WAY-100,635-infused group; ^#^ *P* < .05, ^##^ *P* < .01 vs the Ringer’s solution-infused group.

We next examined whether blockade of the 5-HT_1A_ receptors in the PFC reverses the suppression of the DA responses to handling stress in the citalopram-treated rats. The citalopram-treated rats (10mg/kg/d, s.c. for 14 days) received an infusion of a 5-HT_1A_ receptor antagonist, WAY-100,635 (1 µM), into the PFC. The infusion of WAY-100,635 for 1 to 2 hours did not affect the basal DA levels (90.55±4.44% of the basal levels). Handling stress induced an increase in the DA levels to 190% of the basal levels when WAY-100,635 was infused into the PFC (Figure 3a). The DA levels were significantly higher than those in the citalopram-treated rats that received an infusion of Ringer’s solution into the PFC (group effect, F_(1, 87)_ = 33.4154, *P* < .0001; time effect, F_(9, 87)_ = 5.4623, *P* < .0001; group-time interaction, F_(9, 87)_ = 4.329, *P* = .0001), suggesting that the upregulation of 5-HT_1A_ receptor signaling plays a central role in the suppression of the DA response by long-term citalopram treatment.

The infusion of WAY-100,635 (1 µM) into the PFC of the citalopram-treated rats did not affect the basal NA levels (79.41±10.40% of the basal levels). Handling stress induced an increase in NA levels when WAY-100,635 was infused (Figure 3b). The relative increase in the NA levels at 20 minutes in the WAY-100,635-infused rats tended to be larger than those in the Ringer’s solution-infused rats, although the difference did not reach statistical significance (group effect, F_(1, 70)_ = 0.3577, *P* = .5517; time effect F_(9, 70)_ = 22.196, *P* < .0001; group-time interaction, F_(9, 70)_ = 0.1115, *P* = .0999). These results suggest that long-term citalopram treatment induces a tendency to suppress the NA response during the stressful period by the upregulation of 5-HT_1A_ receptor signaling, but the mechanism is not involved in the decrease in the basal NA levels.

### Effects of Local Infusion of 5-HT_1A_ Receptor Antagonist, WAY-100,635, into PFC of Citalopram-Treated Rats on Immobility Time in FST

To investigate the role of the upregulation of 5-HT_1A_ receptor signaling, which suppresses the DA response to handling stress, in the antidepressant action of citalopram, the effect of an infusion of WAY-100,635 (1 µM) into the PFC on the immobility time in the FST was assessed in the citalopram-treated rats (supplementary Figure 2). Long-term citalopram administration (10mg/kg/d, s.c. for 14 days) decreased the immobility time as expected (1-way ANOVA: F_(2, 27)_ = 12.27, *P* = .0002). The infusion of WAY-100,635 (1 µM) into the PFC for 2 hours prior to the FST did not affect the decreased immobility time in the citalopram-treated rats.

### Effects of Local Infusion of α_2_-Adrenoceptor Antagonist, Idazoxan, into PFC of Citalopram-Treated Rats on DA and NA Levels after Handling Stress

**Figure 4.**
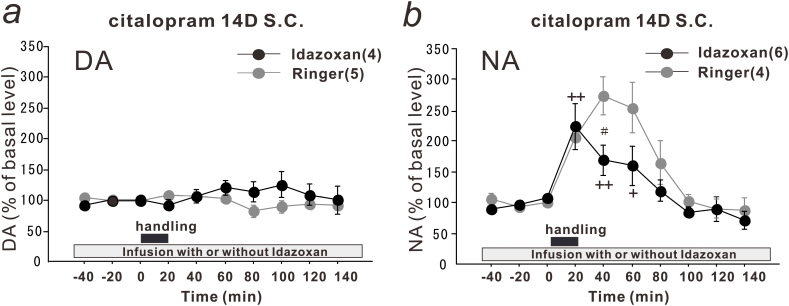
Effects of a local infusion of an α_2_-adrenoceptor antagonist, idazoxan, into the prefrontal cortex (PFC) of citalopram-treated rats on the dopamine (DA) and noradrenaline (NA) levels after handling stress. The citalopram-treated rats received a local infusion of idazoxan (1 µM) (closed circles) into the PFC, and the effects of handling stress on the extracellular levels of DA (a) and NA (b) in the PFC were compared with those in the citalopram-treated rats infused with Ringer’s solution (gray circles, reproduced from Figure 1b and 1d for comparison). The closed squares indicate the 20-minute handling period. All values are calculated as a percentage of the basal values within the same group. The numbers of experiments are shown in parentheses. The data are expressed as the means ± SEM. ^+^ *P* < .05, ^++^ *P* < .01 vs the basal levels of the idazoxan-infused group; *P* < .05 vs the Ringer’s solution-infused group.

We previously reported that chronic citalopram treatment suppresses the NA responses to handling stress in the basolateral amygdala due to sensitization of α_2_-adrenoceptors (Y. Kawahara et al., 2007). To evaluate the contribution of α_2_-adrenoceptors in the PFC, the α_2_-adrenoceptor antagonist idazoxan (1 µM) was infused into the PFC of the citalopram-treated rats. Infusion of idazoxan at 10 and 100 µM has been shown to increase the basal levels of NA and/or DA in the PFC and amygdala up to approximately 200% (Devoto et al., 2001; Ferry et al., 2015), and the high doses of idazoxan may mask the DA and NA responses to acute handling stress. Therefore, the dose of idazoxan at 1 µM, which does not affect the basal levels of DA or NA, was selected in the present study. The infusion of idazoxan for 1 to 2 hours slightly increased the basal DA levels (123.05±4.00% of the basal levels, t(8)=-5.543, *P* = .0005), similar to the previous report (Devoto et al., 2001). The DA levels in the idazoxan-infused rats were not affected by handling stress, as observed in the Ringer’s solution-infused rats (Figure 4a). The results indicate that sensitization of the α_2_-adrenoceptors is not involved in the suppression of the DA responses to handling stress in the PFC of the citalopram-treated rats.

The infusion of idazoxan into the PFC of the citalopram-treated rats did not affect the basal NA levels (94.13±8.74% of the basal levels). When idazoxan was infused, handling stress induced a maximal increase in the NA levels to 230% of the basal levels at 20 minutes in the citalopram-treated rats (Figure 4b). However, at later time points, the idazoxan infusion suppressed the handling stress-induced increase in the NA levels compared with that in the Ringer’s solution-infused rats (group effect, F_(1, 79)_ = 6.285, *P* = .0142; time effect, F_(9, 79)_ = 13.663, *P* < .0001; group-time interaction, F_(9, 79)_ = 1.842, *P* = .0735). These results suggest that the upregulation of α_2_-adrenoceptors is partially involved in the enhancement of the NA response during the recovery period but not during the stressful period or under basal conditions.

## Discussion

The present study demonstrated that acute handling stress induced increases in the extracellular DA and NA levels in the PFC and that both the DA and NA responses to handling stress were negatively regulated by 5-HT_1A_ receptor activation under the control conditions. Long-term treatment with citalopram abolished the DA response without affecting the basal DA levels. The abolishment of the DA response was mediated through upregulation of 5-HT_1A_ receptor signaling. On the other hand, long-term citalopram treatment decreased the basal NA levels, but the NA response was maintained despite the low basal NA levels. 5-HT_1A_ receptor- or α_2_-adrenoceptor–mediated signaling partially affected the NA response. Alteration of DA and NA systems was induced by long-term, but not by acute, citalopram treatment. The differential regulation of the DA and NA systems in the PFC induced by long-term citalopram treatment may be involved in the therapeutic action of antidepressants in depression and anxiety disorders.

### DA Response to Acute Handling Stress in PFC

Acute handling stress induced an increase in the DA levels (the DA response) in the PFC. The DA response is similar to the increases in the DA levels (160%-300% of basal levels) by other acute stressors with various intensities such as restrain (Mokler et al., 2007), injection (Beaufour et al., 2001), tail pinch (Butts et al., 2011), and hypotension (Y. Kawahara et al., 1999). The range of the DA response does not directly reflect the stress intensity as plasma corticosterone.

It has been reported that the DA response to handling stress in the PFC is mediated by glutamatergic signaling in the VTA and PFC (Enrico et al., 1998; Takahata and Moghaddam, 1998; Del Arco and Mora, 2001). In addition, the role of glucocorticoid receptors in the PFC has been demonstrated in the DA response to tail-pinch stress (Butts et al., 2011). The activation of dopaminergic signaling in the PFC by acute social defeat has been implicated as a mechanism for stress resistance (Tanaka et al., 2012). Chronic social defeat suppresses dopaminergic signaling via the prostaglandin E_2_-EP1 signaling pathway, leading to the increased susceptibility to stress and the induction of social avoidance (Tanaka et al., 2012), although contradictory effects of chronic social defeat on dopaminergic signaling have been reported (Cao et al., 2010). Taken together, the DA response to acute stress could be involved in mediating the sensitivity to stress and the subsequent depression-like behaviors.

### A Role for 5-HT_1A_ Receptors in the DA Response to Acute Handling Stress

Activation of 5-HT_1A_ receptors by exogenous agonists has been shown to increase the DA levels under basal conditions (nonstressful conditions) (Rasmusson et al., 1994; Wedzony et al., 1996; Llado-Pelfort et al., 2012). Although 5-HT_1A_ receptors are expressed on both pyramidal neurons and GABAergic interneurons in the PFC (Amargos-Bosch et al., 2004; Santana et al., 2004), 5-HT_1A_ receptor agonists seem to preferentially activate the 5-HT_1A_ receptors on GABAergic interneurons, leading to disinhibition of pyramidal neurons and activation of the mesocortical (VTA-PFC) dopaminergic pathway (Diaz-Mataix et al., 2005; Llado-Pelfort et al., 2012; Celada et al., 2013). In the present study, local infusion of a 5-HT_1A_ receptor agonist, 8-OH-DPAT, into the PFC did not affect the basal DA levels. It is possible that higher doses of 8-OH-DPAT would increase the basal DA levels, although the high doses of 8-OH-DPAT might decrease the basal DA levels, possibly by activating 5-HT_1A_ receptors on pyramidal neurons (Diaz-Mataix et al., 2005). As alteration of the basal DA levels could mask the DA response to acute handling stress, this dose of 8-OH-DPAT, which does not affect the basal DA levels, was selected.

Local infusion of 8-OH-DPAT in the PFC suppressed the DA response to acute handling stress. The effect of 8-OH-DPAT could be explained by preferential activation of the 5-HT_1A_ receptors expressed on the pyramidal neurons. The inhibitory effect of the 5-HT_1A_ receptors on the pyramidal neurons would counteract the stimulatory effect of handling stress, leading to the suppression of the DA response. Thus, the postsynaptic 5-HT_1A_ receptors in the PFC are a key component to regulate the DA system. The switching mechanisms for the preferential activation of the 5-HT_1A_ receptors on the GABAergic interneurons under basal (nonstressful) conditions to that on the pyramidal neurons under acute stressful conditions are not fully understood. The switching could be related to the synaptic plasticity and remodeling of excitatory synapse at pyramidal neurons induced by acute stress (Yuen et al., 2009; Musazzi et al., 2015; Nava et al., 2015). Enhancement of excitatory neurotransmission onto pyramidal neurons by acute stress induces activation of pyramidal neurons, leading to the DA release. Under such conditions, the ability of 5-HT_1A_ receptors to inhibit pyramidal neurons might become dominant compared with the functions of 5-HT_1A_ receptors on GABAergic interneurons and 5-HT_2A_ receptors coexpressed on pyramidal neurons (Celada et al., 2002) and attenuate the DA response to acute stress. The concomitant increase in 5-HT in the PFC in response to acute stress (Fujino et al., 2002) might also contribute to the potentiation of 5-HT_1A_ receptor function on pyramidal neurons.

In this study, 8-OH-DPAT is used as the 5-HT_1A_ receptor agonist. However, 8-OH-DPAT is known to activate with moderate affinity the 5-HT_7_ receptors (Jasper et al., 1997), which are expressed in the PFC (Hoyer et al., 2002), regulate the release of DA and NA in the PFC (Wesolowska and Kowalska, 2008), and play a role in the pathophysiology of anxiety and depression (Hedlund, 2009). The possibility that, in addition to 5-HT_1A_ receptors, 5-HT_7_ receptors are involved in 8-OH-DPAT–induced suppression of the DA and NA responses to acute stress cannot be ruled out.

### Effect of Long-Term Citalopram Treatment on DA Response to Acute Handling Stress

Long-term treatment with citalopram abolished the DA response to acute handling stress by upregulating 5-HT_1A_ receptor signaling. The upregulation of 5-HT_1A_ receptor signaling was pharmacologically proven using the selective 5-HT_1A_ receptor antagonist, WAY-100,635. We did not assess the mechanisms that promote this upregulation. However, chronic citalopram treatment has been shown to increase 5-HT_1A_ receptor agonist-stimulated [^35^S]-GTPγS binding in the PFC and hippocampus (Moulin-Sallanon et al., 2009). In the hippocampus, chronic antidepressant treatment is also shown to enhance the tonic activation of postsynaptic 5-HT_1A_ receptors (Haddjeri et al., 1998). The 5-HT_1A_ receptor protein in the PFC (Szewczyk et al., 2010) or the 5-HT_1A_ receptor binding in the PFC or hippocampus (Welner et al., 1989; Ulrichsen et al., 1992; Moulin-Sallanon et al., 2009; Shrestha et al., 2014) is reported to be unaltered or increased after chronic antidepressant treatment. Such upregulation of 5-HT_1A_ receptor signaling likely contributes to the therapeutic action of antidepressants, since a reduction of the 5-HT_1A_ receptor levels in cortical regions has been observed in animal models of depression and anxiety disorders (Overstreet et al., 2003; Shively et al., 2006) and in patients with depression (Savitz et al., 2009; Stockmeier et al., 2009) and anxiety disorders (Neumeister et al., 2004; Sullivan et al., 2005; Lanzenberger et al., 2007). Our findings support the benefit of partial 5-HT_1A_ receptor agonists, which are selective for postsynaptic 5-HT_1A_ receptors, in the treatment of depression and anxiety disorders (Celada et al., 2013).

Conflicting data on 5-HT_1A_ receptor expression under stressed conditions are reported. In mice, chronic stress (unpredictable chronic mild stress) is shown to increase the 5-HT1_A_ receptor mRNA and protein levels in the cortex, and chronic antidepressant treatment reverses the increased 5-HT_1A_ receptor expression (Le Francois et al., 2015). There are reports showing the increase in 5-HT_1A_ receptor binding in the PFC in patients with depression under specified conditions (Matsubara et al., 1991; Arango et al., 1995) and the reduction of 5-HT_1A_ receptor binding after chronic antidepressant treatment in patients with anxiety disorders (Spindelegger et al., 2009). Taken together, these studies suggest that the effect of antidepressants on 5-HT1_A_ receptor signaling may be dependent on the pathological state where 5-HT_1A_ receptors are dysregulated.

It has been proposed that the HPA axis is an important target of antidepressants, because antidepressants reduce the basal and stimulated HPA axis activity in depressed patients and animals (Pariante et al., 2004). Long-term citalopram treatment might decrease the HPA axis activity and the brain corticosterone levels, resulting in the suppression of the DA response to handling stress (Butts et al., 2011). However, long-term treatment with citalopram at similar doses with this study is shown not to affect the plasma corticosterone response to restraint stress (Hesketh et al., 2005; Garabadu et al., 2015). Therefore, long-term citalopram treatment could suppress the DA response to handling stress without modulation of the corticosterone levels in the PFC.

### A Role for 5-HT_1A_ Receptors in NA Response to Acute Handling Stress in PFC

The acute handling stress-induced increase in the NA levels (the NA response) was suppressed by local infusion of 8-OH-DPAT into the PFC, similar to the suppression of the DA response. Although the evidence for 5-HT_1A_ receptor-mediated regulation of the NA levels in the PFC is limited, similar mechanisms as those used to suppress the DA response would be involved in the NA response. Because pyramidal neurons in the PFC are known to project to the locus coeruleus (LC) (El Mansari et al., 2010; Chandler et al., 2014), the inhibition of pyramidal neurons by the 5-HT_1A_ receptors might suppress the NA response via decreased activation of NA neurons in the LC as well as noradrenergic terminals in the PFC.

The NA system plays an important role in the pathophysiology of neuropsychiatric disorders and is one of therapeutic targets for antidepressants (Pozzi et al., 1994; Linner et al., 2001). Antidepressants that act on both the DA and NA systems have been proposed to further improve the symptoms of depression, particularly in patients who are resistant to treatment (H. Kawahara et al., 2001; Pan et al., 2004; El Mansari et al., 2010). Postsynaptic 5-HT_1A_ receptors, which suppress both the DA and NA responses to handling stress, might be involved in the regulation of stress sensitivity (Savitz et al., 2009).

### Effect of Long-Term Citalopram Treatment on Basal NA Levels

Chronic citalopram treatment is found to decrease the basal NA levels in the PFC. Chronic clomipramine treatment has also been shown to decrease NA contents in the frontal cortex and other brain regions (Adell et al., 1989). However, in a series of analyses using different types of antidepressants, Dazzi et al. (2002a, 2002b, 2003, 2005) could not detect the changes in the basal NA levels in the PFC after chronic treatment. Interestingly, chronic treatment with the NA reuptake inhibitor reboxetiine or desipramine is shown to increase the basal NA levels in the PFC (Page and Lucki, 2002; Higashino et al., 2014). Taking these observations into account, we can hypothesize that antidepressants with properties of the serotonin reuptake inhibitor may decrease the basal NA levels, whereas antidepressants with properties of the NA reuptake inhibitor may increase the basal NA levels in the PFC after chronic treatment. Therefore, it is possible that citalopram with high and selective ability to inhibit serotonin reuptake could induce the decrease in the basal NA levels.

**The remarkable reduction of the basal NA levels in the PFC** (to approximately 40%) **is in agreement with our previous studies in the amygdala** (to approximately 25%) **and** LC (to approximately 45%) **(Y.** Kawahara et al., 2007 **). T**he reduction of the basal NA levels in the PFC, amygdala, and LC is likely associated with a decrease in the spontaneous firing of NA neurons after long-term citalopram treatment, which may be mediated through the enhanced inhibitory function of GABA neurons by 5-HT_2A_ receptors (Szabo et al., 2000; Szabo and Blier, 2001a, 2001b). Alternatively, NA neurons can be inhibited by α_2_-adrenoceptors activated via enhanced NA neurotransmission, which is shown after local infusion of citalopram into the LC (Mateo et al., 2000). However, acute systemic administration of citalopram did not affect the basal NA levels in the present (supplementary Figure 1) and other studies (Umehara et al., 2013). As α_2_-adrenoceptors in the LC **(Y.** Kawahara et al., 2007) or PFC (Figure 4b) are not upregulated under nonstressful conditions, it is unlikely that α_2_-adrenoceptors play a major role in the decrease in the basal NA levels after long-term citalopram treatment. In addition, the 5-HT_1A_ receptors in the PFC might not be involved, because the infusion of the 5-HT_1A_ receptor antagonist in the PFC did not affect the basal NA levels.

### Effect of Long-Term Citalopram Treatment on NA Response to Acute Handling Stress

In contrast to the DA response, t**he NA response to acute handling stress in the PFC was maintained after long-term citalopram treatment, although the basal NA levels were decreased. It is interesting to note that the NA response to acute handling stress in the amygdala was abolished** after long-term treatment with citalopram**, whereas the NA response** in the LC was maintained **(Y.** Kawahara et al., 2007). **Thus, the NA response to acute stress seems to be heterogeneously regulated in various brain regions; the NA response in the PFC resembles that in the LC, but not in the amygdala.**

**5-HT**_**1A**_ **receptor blockade in the PFC induced a tendency to increase the NA response to acute handling stress during the stressful period but not the recovery period. It is possible that the upregulation of 5-HT**_**1A**_ **receptor signaling leads to partial suppression of the NA response. However, the contribution of upregulated 5-HT**_**1A**_ **receptor signaling to the NA response is much smaller than that to the DA response.**

**In agreement with our observations,** Dazzi et al. (2005 **) have reported that** chronic **treatment with a SSRI, fluvoxamine, does not alter the NA response to acute foot shock stress. On the other hand, chronic treatment with fluoxetine (SSRI) is reported to enhance the NA response to stress in the hippocampus, possibly due to downregulation of 5-HT**_**1A/1B**_ **receptors on LC neurons** (Page and Abercrombie, 1997 **). In our study,** 5-HT_1A_ receptors **in the PFC are rather upregulated after chronic citalopram treatment. Based on these findings, one can hypothesize that functional states of** 5-HT_1A_ receptors after chronic SSRI treatment may be an important regulator of the NA response to acute stress.

**Other types of antidepressants with properties to enhance** NA neurotransmission **(e.g., reboxetine, venlafaxine, and mirtazapine) are shown to suppress the NA response to acute stress in the PFC** (Dazzi et al., 2002b, 2002a, 2003 **), although the opposing effects of chronic reboxetine treatment are reported in its continuous presence** (Page and Lucki, 2002 **). Thus, SSRIs likely maintain the responsiveness of the NA to acute stress with the decrease in the basal NA levels, whereas antidepressants acting on the NA system suppress the NA response with the increase in the basal NA levels. Accordingly, antidepressants acting on both 5-HT and NA systems such as tricyclic antidepressants and serotonin and noradrenalin reuptake inhibitors seem to induce the alterations of the NA response to acute stress and the basal NA levels depending on the balance of properties to enhance 5-HT and NA neurotransmission.**

We previously reported that long-term citalopram treatment sensitizes the α_2_-adrenoceptors in the amygdala, which likely contributes to the attenuation of the NA response to handling stress (Y. Kawahara et al., 2007). In this study, contrary to our expectation, blockade of the α_2_-adrenoceptors attenuated the NA response to handling stress in the PFC. An electrophysiological study demonstrated that activation of the postsynaptic α_2_-adrenoceptors in the PFC hyperpolarizes GABAergic interneurons and thereby disinhibits the pyramidal cells (Andrews and Lavin, 2006). Therefore, it is possible that the blockade of the α_2_-adrenoceptors in the PFC may inhibit the pyramidal cells and attenuate the NA response to handling stress.

## Conclusion

Chronic treatment with antidepressants is demonstrated to reduce the basal NA levels and abolish the DA response to acute stress in the PFC. The suppression of the DA response is mediated through upregulation of the postsynaptic 5-HT_1A_ receptors. The differential regulation of the DA and NA systems in the PFC may be involved in the therapeutic action of citalopram. Furthermore, this study provides the basis for the therapeutic action of partial 5-HT_1A_ receptor agonists in psychiatric disorders.

## Statement of Interest

None.

## Supplementary Material

supplementary Table 1
