# Supplementary material for: Long-Term Citalopram Treatment Alters the Stress Responses of the Cortical Dopamine and Noradrenaline Systems: the Role of Cortical 5-HT1A Receptors
Source: Int J Neuropsychopharmacol. 2016 Mar 30;19(8):pyw026. doi: 10.1093/ijnp/pyw026 (PMC5006198; doi:10.1093/ijnp/pyw026)
Supplement: supplementary Table 1 [file Supplementary_Figure_S2.docx]

Supplementary Figure S2
